# Using smartphone-based assessment of social experience to predict relapse in first-episode psychosis

**DOI:** 10.1038/s41537-026-00786-3

**Published:** 2026-07-17

**Authors:** Ryan Hammoud, Anna Georgiades, Maria Chiara Del Piccolo, Shangqing Liu, Aljawharah Almuqrin, Robert Stewart, Ioannis Bakolis, Natasha Vorontsova, Stefania Tognin, Andrea Mechelli

**Affiliations:** 1 Department of Psychosis Studies, Institute of Psychiatry, Psychology & Neuroscience. King’s College London, London, UK; 2 Central and North West London NHS Foundation Trust, Brent Early Intervention Service, London, UK; 3 College of Artificial Intelligence Medicine, Chongqing Medical University, Chongqing, China; 4 Department of Health Sciences, College of Health and Rehabilitation Sciences, Princess Nourah bint Abdulrahman Univeristy, Riyadh, Saudi Arabia; 5 Department of Psychological Medicine, Institute of Psychiatry, Psychology & Neuroscience, King’s College London, London, UK; 6 South London and Maudsley NHS Foundation Trust, London, UK; 7 Centre for Mental Health Policy & Evaluation. Health Service & Population Research Department. Institute of Psychiatry, Psychology & Neuroscience, King’s College London, London, UK

**Keywords:** Human behaviour, Psychosis

## Abstract

Social-affective changes are early indicators of psychosis relapse, yet their dynamic and subjective nature makes them difficult to capture between routine clinical assessments. Smartphone-based ecological assessments offer a method for capturing real-time social experiences in daily life. To address this gap, we examined whether social contact and social experience, measured through smartphone-based assessments, were associated with relapse following a First Episode of Psychosis (FEP), and evaluated whether smartphone data could predict relapse using machine learning. This longitudinal study included 256 individuals with FEP who completed at least one smartphone assessment, recruited from 10 Early Intervention Services across England and Wales between 2020–2023. Participants completed baseline clinical assessments and up to 30 days of daily smartphone surveys assessing social contact (alone vs with others) and social experience (a composite measure of emotional response to social contact). Relapse was assessed via structured interviews and electronic health records at 4, 8, and 12 months. Of 256 participants analysed (mean age 25.7 years [SD 5.3], 44.5% female), 8.2%, 13.5%, and 20.1% relapsed within 4, 8, and 12 months. Higher social experience consistently predicted reduced relapse risk across follow-ups, with similar effects for daily and momentary measures, while social contact showed inconsistent associations. Machine learning models using five smartphone assessments within 30 days achieved a validation accuracy of 0.790 (AUC 0.829) for predicting 12-month relapse, while data from a single smartphone assessment within 7 days achieved an accuracy of 0.715. These findings suggest that brief smartphone-based self-reports of social experience provide a low-burden, scalable tool for detecting early relapse risk and supporting more timely, personalised interventions in psychosis care.

## Introduction

Psychotic disorders are among the most severe mental health conditions, with up to 80% of individuals experiencing a relapse within five years^1^. Early identification of relapse risk is essential to improving long-term outcomes. Among the potential early indicators of relapse, disruptions in social experience and functioning, are particularly relevant^2–4^. Social withdrawal^5,6^, loneliness^7,8^, reduced social motivation^2^, and altered affective responses to others^9^ have been linked to symptom exacerbation and increased risk of relapse. These changes often precede the emergence of psychosis symptoms and interfere with a person’s ability to cope or seek help, making them critical targets for early detection.

Routine care after a FEP typically involves time-limited specialist services^10,11^ and relies on periodic clinical assessments^12^. While these can capture symptom severity during clinical contact, they cannot monitor the rapid changes that often occur between visits. The effective monitoring of these changes may be further hindered by reduced insight, cognitive deficits, and difficulties recalling early symptom changes in this clinical population^13,14^. In-person assessments are also resource-intensive and challenging to scale, especially in models of care that emphasise low caseloads^15^. These limitations underscore the need for practical, real-time tools that can be implemented widely for monitoring relapse risk.

Smartphone-based assessments offer a cost-effective and ecologically valid approach by capturing symptoms in real time and reducing reliance on retrospective recall^16^. They can detect early indicators of relapse between clinical visits thereby supporting more responsive care^17,18^, and are highly scalable and minimally intrusive. Recent studies show that 90% of individuals with psychosis own a smartphone, supporting its feasibility in clinical populations^19^, and that smartphone-based monitoring is broadly acceptable in FEP populations^20^. Emerging evidence has shown that changes in social behavior captured via smartphones, often precede relapse, with prediction models showing promising accuracy^12,21,22^. However, these models have generally relied on small cohorts with few relapse events, limiting their robustness and generalisability. Most have relied on passive sensing data (e.g., GPS location patterns, accelerometer, and estimated sleep patterns), and on basic phone usage metrics (e.g., calls, texts) as proxies for social behavior, rather than directing assessing social-affective experiences. While these approaches can capture broad behavioral shifts, they may overlook the subjective experiences of social interactions that could provide more specific indicators of relapse risk.

The current study aimed to examine whether social contact (the objective presence or absence of others) and social experience (an individual’s subjective emotional response to their social environment) are associated with relapse risk in individuals following a FEP. Specifically, by monitoring these experiences in real-time, we aimed:

1. To examine whether social contact, being alone versus being with others, over a 30-day period is associated with risk of first relapse following the initial episode of psychosis within 4, 8, and 12 months following a baseline clinical assessment.
2. To examine whether social experience, the subjective emotional response to social context, over a 30-day period is associated with risk of first relapse following the initial episode of psychosis relapse within 4, 8, and 12 months.
3. To evaluate the accuracy of machine learning models using smartphone-based data to predict first relapse following the initial episode of psychosis within 12 months at the individual level, and to examine how predictive performance varies with the number of assessments.

While the first two aims focus on explanatory analyses to examine associations between social processes and relapse, the third aim adopts a predictive modelling approach to evaluate the extent to which these data can be used to forecast relapse at the individual level.

We hypothesised that being alone would be associated with greater relapse risk than being with others within 4-, 8-, and 12-month follow-up (Hypothesis 1). We also hypothesised that negative social experience would be associated with increased risk of psychosis relapse across the same time points (Hypothesis 2). Finally, we hypothesised that machine learning models using smartphone-based data would predict psychosis relapse at the individual level with statistically significant accuracy, and that predictive performance would improve with a greater number of assessments and monitoring days (Hypothesis 3).

## Results

### Sample characteristics

A total of 274 participants were recruited in the Social Mind study. Of these, 18 were excluded from the current analyses: 13 participants did not complete any smartphone-based assessments, and 5 withdrew consent and requested that their data be removed. The final sample included 256 participants who downloaded the Social Mind app and completed at least one smartphone-based assessment. Socio-demographic characteristics are reported in Table 1.

**Table 1:** Sociodemographic and clinical characteristics of the full study sample and the subsample providing smartphone-based assessments.

		Full sample (*n* = 269)	Smartphone sample (*n* = 256)
**Age, mean (SD)**		25.56 (5.28)	25.68 (5.25)
**Range**		18–40	18–40
**Gender (%)**	Female	118 (43.86)	114 (44.53)
	Male	151 (56.13)	142 (55.47)
**Ethnicity (%)**	White	100 (37.17)	92 (35.94)
	Black	75 (27.88)	73 (28.52)
	Asian	45 (16.73)	44 (17.19)
	Other	49 (18.22)	47 (18.36)
^**a**^ **Employment (%)**	Employed	135 (51.92)	129 (52.23)
	Unemployed	125 (49.08)	118 (47.77)
^**a**^ **Student Status (%)**	In education	73 (27.34)	67 (26.38)
	Not in education	194 (72.66)	187 (73.62)
^**a**^ **Education Level (%)**	Less than high school	13 (4.89)	11 (4.35)
	High school	131 (49.25)	125 (49.41)
	Professional training	28 (10.53)	24 (9.49)
	University	76 (28.57)	75 (29.64)
	Post-graduate	18 (6.77)	18 (7.11)
^**b**^ **Completed smartphone assessments per participant, mean (SD)**			13.75 (8.92)
^**a**^ **Psychosis Relapse**	Within 4 months		
	Yes	22 (8.59)	20 (8.16)
	No	234 (91.41)	225 (91.84)
	Within 8 months		
	Yes	35 (14.11)	32 (13.50)
	No	213 (85.89)	205 (86.50)
	Within 12 months		
	Yes	50 (20.41)	47 (20.09)
	No	195 (79.59)	187 (79.91)
^**a**^ **Hallucinations**^**†**^	Within 4 months		
	Yes	44 (21.57)	43 (21.61)
	No	160 (78.43)	156 (78.39)
	Within 8 months		
	Yes	57 (30.00)	55 (29.57)
	No	133 (70.00)	131 (70.43)
	Within 12 months		
	Yes	65 (33.51)	63 (33.33)
	No	129 (66.69)	126 (66.67)
^**a**^ **Delusions**^**†**^	Within 4 months		
	Yes	48 (23.88)	45 (22.96)
	No	153 (76.12)	151 (77.04)
	Within 8 months		
	Yes	69 (36.90)	66 (36.07)
	No	118 (63.10)	117 (63.93)
	Within 12 months		
	Yes	79 (40.51)	76 (40.00)
	No	116 (59.49)	114 (60.00)

^a^Variations in total counts are due to incomplete data or participant dropout.

^b^Maximum possible number of assessments was 30 (daily prompts for 30 days).

^†^Hallucinations and delusions were derived from the SCID-IV Psychosis Module and coded as cumulative binary indicators across follow-up. These variables were included for sample characterisation only and were not used as predictors in the analyses.

### Association between social contact and relapse

Logistic regression models showed inconsistent associations between daily and momentary social contact and risk of relapse within 4, 8, and 12 months (see Table 2). In the main analysis models using smartphone data collected within 30 days of baseline, daily social contact (being alone vs. with others over the past 24 h) was significantly associated with greater odds of relapse within 4 months (OR: 2.33; 95% CI: 1.09, 4.98), but not within 8 or 12 months. In the 7-day sensitivity analyses, associations were significant across all time points, while in the 15-day models, associations were significant within 4 months (OR: 2.54; 95% CI: 1.30, 4.96) and 8 months (OR: 1.97; 95% CI: 1.00, 3.87), but not within 12 months.

**Table 2:** Associations between being alone (vs. with others) and relapse within 4, 8, and 12 months, with sensitivity analyses using data from 7-, 15- and 30-day windows after baseline.

		Relapse		
		Within 4 months	Within 8 months	Within 12 months
		OR 95% CI	OR 95% CI	OR 95% CI
7 days of smartphone data	Daily social contact	3.30** (1.56, 7.00)	2.69** (1.33, 5.44)	2.26* (1.12, 4.55)
	Momentary social contact	1.75 (0.94, 3.26)	1.36 (0.84, 2.21)	1.29 (0.88, 1.89)
15 days of smartphone data	Daily social contact	2.54** (1.30, 4.96)	1.97* (1.00, 3.87)	1.94 (0.97, 3.90)
	Momentary social contact	1.68 (0.86, 3.27)	1.38 (0.83, 2.30)	1.49 (1.00, 2.23)
30 days of smartphone data	Daily social contact	2.33* (1.09, 4.98)	1.90 (0.90, 4.01)	1.88 (0.88, 4.02)
	Momentary social contact	1.75 (0.88, 3.47)	1.57 (0.93, 2.64)	1.68* (1.10, 2.56)

*p < 0.05, **p < 0.01.

Analyses were adjusted for age, gender, ethnicity, education level, employment and student status.

In the main 30-day models, momentary social contact (being alone vs. being with others at the time of assessment) was significantly associated with greater odds of relapse within 12 months (OR: 1.68; 95% CI: 1.10, 2.56), but not within 4 or 8 months. Momentary social contact was not significantly associated with relapse in the 7- or 15-day models.

### Association between social experiences and relapse

**Fig. 1:**
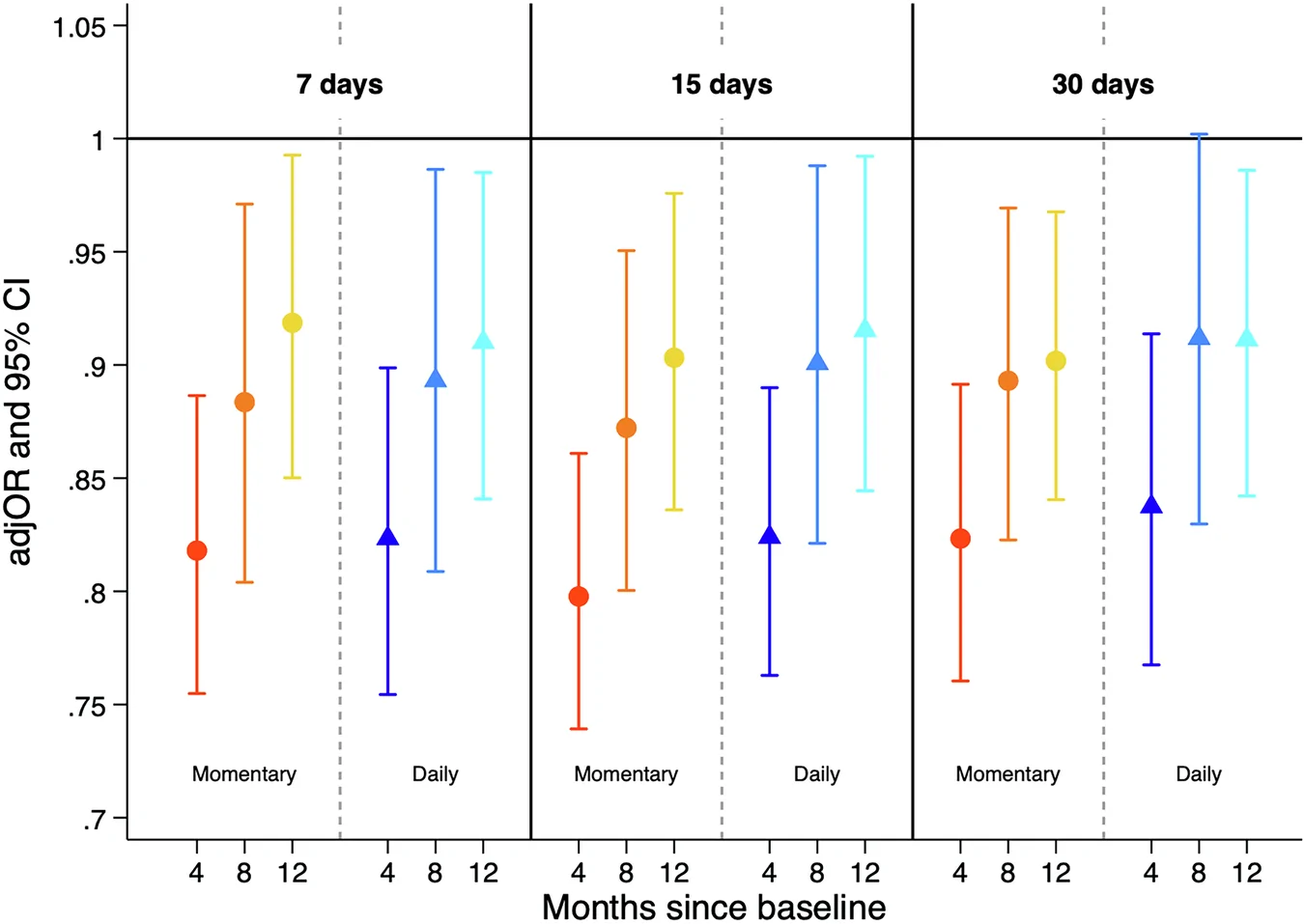
Association between momentary and daily social experience and relapse within 4, 8, and 12 months, using data collected over 7-, 15-, and 30-days post-baseline. All models were adjusted for age, gender, ethnicity, education level, employment and student status. Plotted values represent adjusted odds ratios (adjOR) and 95% confidence intervals.

Consistent associations were observed between higher momentary and daily social experience scores, reflecting more positive social experience, and reduced risk of relapse within 4, 8 and 12 months (See Fig. 1). In models using data collected over the first 30 days post-baseline, higher daily social experience scores were significantly associated with lower odds of relapse within 4 months (OR: 0.84; 95% CI: 0.77, 0.91) and 12 months (OR: 0.91; 95% CI: 0.84, 0.99), while the association at 8 months trended in the expected direction, but did not reach statistical significance (OR: 0.91; 95% CI: 0.83, 1.00). Daily social experience was significantly associated with reduced relapse risk across all time points in the 7- and 15-day sensitivity models.

Similarly, higher momentary social experience scores were also significantly associated with reduced odds of relapse at all timepoints in the 30-day models (OR: 0.82; 95% CI: 0.76, 0.89 within 4 months; OR: 0.89; 95% CI: 0.82, 0.97 within 8 months; OR: 0.90; 95% CI: 0.84, 0.97 within 12 months). This pattern was consistent in the 7- and 15-day sensitivity analyses (See Fig. 1). All models were adjusted for age, gender, ethnicity, employment status, and education, and results were also consistent in unadjusted analyses (See Suppl. Table 2).

### Prediction of psychosis relapse using social experience data and machine learning models

**Fig. 2:**
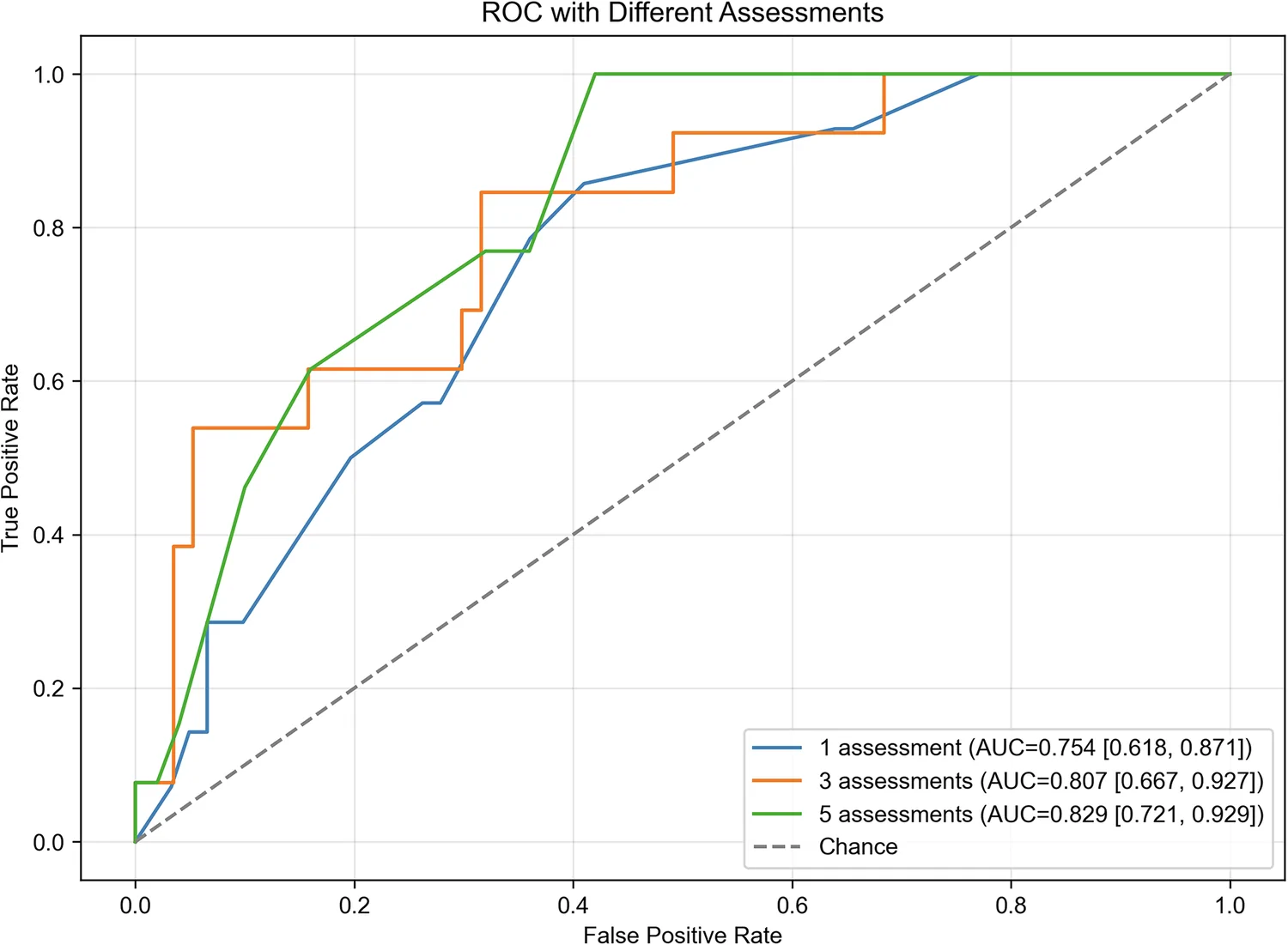
ROC curves for machine learning models using 1, 3, and 5 assessments collected within 30 days post-baseline to predict 12-month psychosis relapse. AUC values and 95% confidence intervals are reported in the legend.

The CatBoost models trained on both social experience and contact data were used to predict relapse within 12 months. Predictive performance varied on the length of the data collection window (7-, 15-, and 30-days post-baseline) and the number of assessments included. Using a single assessment collected within the first 7 days, the model achieved a validation accuracy of 0.715 [0.554, 0.855] (*p* = 8.457e-6) and AUC of 0.707 [0.566, 0.845] (*p* = 5.196e-3), indicating moderate predictive performance (See Suppl. Table 4). Utilising three assessments within 15 days yielded a validation accuracy of 0.788 [0.634, 0.829] (*p* = 3.829e-12) and AUC of 0.772 [0.667, 0.871] (*p* = 2.974e-5)). The highest performance was observed with a model trained on 5 assessments over a data collection window of 30 days post-baseline, achieving a validation accuracy of 0.790 [0.721, 0.860] (*p* = 0.011) and AUC of 0.829 [0.721, 0.929] (*p* < 7.97e-10) (See Fig. 2 and Table 3).

**Table 3:** Validation performance of CatBoost models for predicting psychosis relapse within 12 months.

Model	AUC	Balanced ACC	sensitivity	specificity	PPV	NPV
1 assessment	0.754 [0.618-0.871]	0.725 [0.600–0.826]	0.856 [0.647–1.000]	0.593 [0.474–0.714]	0.326 [0.176–0.485]	0.947 [0.871–1.000]
3 assessments	0.807 [0.667-0.927]	0.742 [0.600–0.882]	0.537 [0.267–0.818]	0.947 [0.877–1.000]	0.700 [0.400–1.000]	0.901 [0.825–0.967]
5 assessments	0.829 [0.721-0.929]	0.790 [0.721, 0.860]	1.000 [1.000–1.000]	0.580 [0.442–0.708]	0.384 [0.226–0.549]	1.000 [1.000–1.000]

Values are presented as mean [95% bootstrap confidence interval; area under the curve (AUC), balanced accuracy, sensitivity, specificity, positive predictive value (PPV), and negative predictive value (NPV) are reported for models using one, three, and five assessments.

## Discussion

In this longitudinal study of FEP individuals, the relationship between daily and momentary social contact and experience and relapse was examined over a 12-month period using smartphone-based assessments.

With regards to the first hypothesis, only partial support was found for an association between social contact and relapse. Being alone over the past 24 h was associated with increased relapse risk within 4 and 8 months, but not consistently within 12 months or across different data collection windows. Momentary social contact (i.e., being alone at the time of assessment) showed even less evidence of an association. These mixed results suggest that the presence of others may not reliably indicate vulnerability to relapse. This contrasts somewhat with prior work linking social withdrawal and isolation to psychosis risk^5,6^, and highlights the potential importance of emotional and contextual dimensions beyond the mere presence of others. The quality of social relationships may be critical, where supportive, validating interactions can be protective, consistent with expressed emotion (EE) literature showing higher relapse in high-EE households vs low-EE households^23^.

Consistent with this study’s second hypothesis, subjective experiences of social contexts over the past 24 h were generally associated with reduced relapse risk within follow-up time points. Greater positive social experience, regardless of whether participants were alone or with others, was linked to lower odds of relapse across most follow-up periods. Similar associations were found for momentary subjective experiences of social context, suggesting that even snapshots of social experience could be sufficient for predicting risk over time. These findings align with prior research suggesting that social-affective disruptions, such as perceived loneliness^7,8^ and altered emotional responses to social contact^2,9,24^ often precede the exacerbation of psychosis symptoms. In addition, these findings extend the existing literature by showing that these dimensions of social life, when measured in real-time for a period as little as 7 days, are robust indicators of future relapse.

Consistent with this study’s third hypothesis, it was found that machine learning models trained on social experience data could predict relapse at the individual level with moderate to strong internal predictive performance. Models trained on five assessments within a 30-day window post-baseline achieved a validation accuracy of 0.790 and an AUC of 0.829. Importantly, even models based on fewer assessments or shorter time windows (e.g., 7 days) showed moderate to strong accuracy. These results suggest that smartphone-based assessments could generate individualised predictions of relapse risk. Taken together, these findings emphasise that subjective social-affective experience may represent a more clinically meaningful signal of relapse vulnerability than objective social exposure alone.

This study has several strengths. First, the sample was relatively large and demographically diverse, recruited from ten early intervention services across England and Wales. Second, the use of smartphone-based assessments enabled the capturing of participants’ experiences in real-time and in real-world contexts, reducing recall bias and enhancing ecological validity. Third, these repeated, low-burden assessments enabled the monitoring of dynamic changes in social environments and of subjective experiences with minimal disruption to one’s daily life. Lastly, the utilisation of both traditional statistical models and advanced techniques of machine learning approaches strengthened the robustness of the findings, enabling both group- and individual-level inferences.

Some limitations should be considered. Variability in the compliance and completion rates may have introduced bias, particularly if non-completion may be associated with relapse risk, as individuals experiencing early signs of relapse may have been less likely to complete assessments. This could result in non-random missing data, potentially biasing both regression estimates and machine learning models, leading to overestimation of predictive performance among more adherent participants. It is also possible that some participants who dropped out subsequently experienced relapse events that were not captured. Relapse classification relied on clinical interviews and electronic health records, but some relapse events may not have been captured if participants were lost to follow-up and disengaged from their care team or were no longer under the care of one of the Early Intervention Services participating in the study. While the sample was drawn from ten NHS services, the requirement for participants to own a smartphone, have occasional access to the internet, and engage with app-based monitoring may have resulted in a more digitally engaged subgroup of individuals with FEP, thereby limiting generalisability to healthcare systems outside the UK and to individuals with lower digital access, lower engagement, or more severe illness who may be less able to or willing to use such technologies. Information on housing status or living arrangement was not collected as part of the study. This is a significant limitation because living with family, partners, or others may influence both social contact and social experience ratings, as well as relapse risk through mechanisms such as social support, stress, expressed emotion, or help-seeking. Residual confounding by living arrangement therefore cannot be excluded, and future studies should collect and adjust for housing status when examining associations between social experience and relapse. Although all participants were required to have experienced an FEP within the previous 24 months, exact duration since first episode, duration of untreated psychosis, and duration in care were not consistently available in the research dataset. We were therefore unable to describe or adjust for these variables, and residual confounding by differences in illness duration or service exposure cannot be excluded. Additionally, although the predictive models showed strong internal performance, they were not externally validated, and their generalisability to independent samples remains to be established. Furthermore, predictors derived from different time windows (e.g., 7-, 15-, and 30-day periods) were partially overlapping, resulting in temporal dependence across analyses. As such, findings across models should not be interpreted as independent replications, but rather as complementary evidence reflecting similar underlying data. Finally, social contact was assessed using brief single-item smartphone questions (“Who is with you right now?” and “Who have you been with in the past 24 h?”), and social experience was measured using purpose-designed items rather than validated measures of social functioning or social networks. While this low-burden approach was intended to facilitate repeated real-time assessment via smartphone, it may have captured only a narrow, context-dependent aspect of social experience rather than broader constructs such as social functioning. A related consideration is that, although participants could indicate whether the people they were with were friends, family members, partners, colleagues or strangers, social experience ratings were not collected separately for each selected relationship type. Instead, these ratings reflected the overall social context. Therefore, we were unable to distinguish whether social experience differed according to the type of relationship, or whether different types of relationships were associated with different levels of relapse risk. Future research would benefit from incorporating validated measures alongside relationship-specific ratings of social experience.

### Clinical implications

This study demonstrates that real-time social experience data, collected via smartphones in everyday contexts, can be used to estimate psychosis relapse within a one-year period with an accuracy of up to 79%. While accuracy of prediction increases with the number of smartphone-based self-reports, even a single assessment collected within the first week allowed an accuracy of 72%. These findings suggest that subtle, momentary shifts in how an individual feels in their social environments offer important signals of clinical vulnerability.

Smartphone-based self-reports are low burden, cost-effective, and can be easily integrated into daily life. While these findings highlight the potential of brief digital assessments to support relapse monitoring, the predictive performance observed here should be interpreted with caution, as further work is needed to evaluate model calibration, sensitivity-specificity trade-offs, and clinically meaningful decision thresholds. Incorporating brief digital assessments of subjective social experience into Early Intervention Services could enhance relapse monitoring between appointments and support more responsive care. However, future research is needed to determine how such tools can be effectively implemented, integrated into care pathways, and externally validated across settings. Ultimately, approaches that prioritise subjective social-affective experience may support more timely, tailored, and accessible relapse prevention strategies for individuals recovering from a first episode of psychosis.

## Methods

Ethical approval for the study was granted by the London-Surrey Research Ethics Committee (20/LO/0331). All participants provided informed consent, and all procedures were conducted in accordance with relevant ethical guidelines and regulations.

### Design and procedures

This prospective, longitudinal observational study assessed relapse risk in FEP using data from the Social Mind smartphone application (Android and iOS), designed to capture daily social experiences linked to relapse vulnerability. The app was developed on the Citizen Scientist platform, allowing researchers to create customised smartphone apps for data collection (https://citizenscientist.app/).

Participants completed structured assessments at baseline and within 4-, 8-, and 12-month follow-ups, conducted remotely or in person. Clinical measures included the Positive and Negative Syndrome Scale (PANSS) for psychotic symptoms^25^, the Hamilton Rating Scale for Depression (HAM-D)^26^, and functional outcomes via the Global Assessment of Functioning (GAF) and Global Functioning Role and Social scales (GF: Role, GF: Social)^27,28^. Participants received £40 per completed assessment. Data were pseudonymised using unique study IDs. Relapse outcomes were determined at the 4-, 8-, and 12-month follow-up assessments using structured interviews and electronic health records.

Following the baseline assessment, participants were onboarded to the Social Mind app. Smartphone-based self-reports were collected during the 30-day period immediately following baseline to capture early post-baseline social experiences. During onboarding, participants selected their preferred time of day to receive a daily notification. Entries could be completed at any point within the subsequent 24 h of receiving a prompt, before the next notification was delivered. Each entry (∼2–3 min), included questions on sleep, social contact, and subjective social experiences, such as perceived enjoyment or stress when alone or with others. Additionally, geolocation and passive activity metrics (e.g., step count, distance travelled) were recorded automatically.

### Participants

Between December 2020 and December 2023, 274 individuals were recruited from 10 Early Intervention Services (EIS) for psychosis across England and Wales. Eligible participants were aged 18–40 years and had experienced an FEP within the previous 24 months. FEP was identified by treating clinical times in routine care using International Classification of Diseases (ICD)-based diagnostic frameworks, and diagnostic eligibility for the study was confirmed using the Structured Clinical Interview (SCID) for DSM-IV, as specified in the study protocol. Eligible DSM-IV diagnoses included schizophrenia, schizophreniform disorder, schizoaffective disorder, or delusional disorder^29^. Additional criteria included smartphone ownership with mobile data access and willingness to complete daily smartphone-based assessments and clinical interviews.

Exclusion criteria were > 1 prior episode of psychosis, inability to provide informed consent, refusal of smartphone or clinical assessment, estimated IQ < 70, or detention under the Mental Health Act 1983 at recruitment.

### Measures

#### Outcome

The primary outcomes were binary indicators of whether a participant experienced a first relapse following the initial episode of psychosis within 4, 8, or 12 months following the baseline assessment. These follow-up intervals were selected a priori to capture relapse across the first year following a first episode of psychosis, a clinically important period during which relapse risk is elevated^1^. For each interval, relapse was coded as yes/no, based on structured clinical interviews and electronical health records. Relapse was defined according to the SCID criteria as re-emergence or worsening of psychosis symptoms requiring clinical intervention. Relapse status was determined by trained researchers at each participating NHS site using all available clinical information. Assessors were blind to smartphone app data, which were only analysed after completion of follow-up assessments. Uncertain cases were resolved through team discussion to reach consensus.

#### Smartphone-based predictors

The main predictors were smartphone-based daily social contact and social experience. In each assessment, participants indicated whether they had been alone or with others in the previous 24 h and rated five items about that context (e.g., relaxing, enjoyable, stressful, upsetting, would prefer not to be alone or with the person or people present) on a 5-point Likert scale. Negative items were reverse scored and summed to create a social experience score (range: 5–25), with higher scores indicating more positive social experience. Secondary smartphone-based predictors were momentary social contact and social experience, which were framed around interactions at the time of the assessment rather than over the past 24 h. These were explored as potentially less prone to recall bias than daily ratings.

#### Confounders

Baseline sociodemographic variables included age, gender, ethnicity (White, Black, Asian, Other), education (less than high school, high school, professional training, university, postgraduate), employment (employed vs. not), and student status (yes/no).

### Statistical analysis

To assess the predictive utility of early smartphone-based data, primary analyses included social contact and experience scores reported within the first 30 days of app use. All smartphone assessments completed within the specified 30-day observation window were included in the analyses. This window balanced feasibility of early monitoring with sufficient data for estimation. Sensitivity analyses were conducted using shorter (7-day and 15-day) windows, which included only assessments completed within the corresponding post-baseline period. Analyses were conducted across multiple time windows (7-, 15-, and 30-day) and follow-up periods (4, 8, and 12 months) to examine the robustness of findings. Given the exploratory nature of these comparisons, results were interpreted with emphasis on consistency of effects across models, rather than isolated statistically significant findings.

Logistic regression models were used to examine associations between four smartphone-derived predictors: momentary social contact (alone vs with others at the time of assessment), momentary social experience (a composite measure of emotional response to social contact at the time of assessment), daily social contact (reports of who participants had been with over the previous 24 h), and daily social experience (a composite score derived from ratings of social experiences over the previous 24 h), and relapse within 4-, 8-, and 12-month follow-ups. Social contact and social experience variables were analysed at the level of individual smartphone assessments. Robust standard errors clustered by participant were used to account for the non-independence of repeated assessments contributed by the same participant. Analyses were first univariate, followed adjusted for age, gender, ethnicity, education, employment, and student status. Associations were reported as odds ratios (ORs) with 95% confidence intervals (CIs). All statistical tests were two-sided. Statistical significance was defined as *P* < 0.05. All analyses were conducted using Stata/MP 18.

### Machine learning model development

Data from the 7-, 15-, and 30-day post-baseline assessment windows were split into training (70%) and testing (30%) sets. Participants were required to complete a minimum number of smartphone assessments within each observation window to be included in each model. Within the 30-day window, separate models were developed for participants completing at least 1, 3, or 5 assessments; within the 15-day window, models were developed for participants completing at least 1 or 3 assessments; and within the 7-day window, participants were required to complete at least 1 assessment. Once participants met the minimum assessment threshold, all available smartphone data collected within the relevant observation window were included in model development. Average daily social contact and social experience scores, along with baseline sociodemographic variables were used as model inputs. Because relapse within 12 months was relatively infrequent (approximately 4 non-relapse to 1 relapse), the Synthetic Minority Oversampling Technique (SMOTE)^30^ was applied to the training set to address class imbalance. Models were developed using gradient-boosted decision trees (CatBoost) and evaluated on testing set using balanced accuracy and the area under the receiver operating characteristic (ROC) curve (AUC), sensitivity, specificity, positive predictive value (PPV), and negative predictive value (NPV). Bootstrap confidence intervals were estimated for each performance metric (See Fig. 1 and Table 3). Further details on preprocessing, model specification, and evaluation procedures are provided in Supplementary Note 1.

## Supplementary information

Supplementary Material

## Data Availability

The data generated and analysed for the current study are not publicly available in accordance with ethical and institutional guidelines, but de-identified data may be made available from the corresponding author on reasonable request.

## Code availability

The analytical code is not publicly available because it was developed using a restricted pseudonymised dataset governed by ethical approvals and data-sharing agreements; however, relevant code excerpts may be made available from the corresponding author upon reasonable request.

## References

[1] Alvarez-Jimenez, M. et al. Risk factors for relapse following treatment for first episode psychosis: A systematic review and meta-analysis of longitudinal studies. *Schizophr. Res.* **139**, 116–128 (2012). 10.1016/j.schres.2012.05.007 22658527

[2] Moe, A. M., Weiss, D. M., Pine, J. G., Wastler, H. M. & Breitborde, N. J. K. Social motivation and behavior in first-episode psychosis: Unique contributions to social quality of life and social functioning. *J. Psychiatr. Res.* **144**, 441–447 (2021). 10.1016/j.jpsychires.2021.11.001 PMC8667854 34749220

[3] Bentall, R. P. & Fernyhough, C. Social Predictors of Psychotic Experiences: Specificity and Psychological Mechanisms. *Schizophr. Bull.* **34**, 1012–1020 (2008). 10.1093/schbul/sbn103 PMC2632492 18703667

[4] Addington, J. et al. The Role of Cognition and Social Functioning as Predictors in the Transition to Psychosis for Youth With Attenuated Psychotic Symptoms. *Schizophr. Bull.* **43**, 57–63 (2017). 10.1093/schbul/sbw152 PMC5216866 27798225

[5] Dragt, S. et al. Environmental factors and social adjustment as predictors of a first psychosis in subjects at ultra high risk. *Schizophr. Res.* **125**, 69–76 (2011). 10.1016/j.schres.2010.09.007 20884179

[6] Mason, O. et al. Risk factors for transition to first episode psychosis among individuals with ‘at-risk mental states’. *Schizophr. Res.* **71**, 227–237 (2004). 10.1016/j.schres.2004.04.006 15474894

[7] Winkler, K., Lincoln, T. M., Wiesjahn, M., Jung, E. & Schlier, B. How does loneliness interact with positive, negative and depressive symptoms of psychosis? New insights from a longitudinal therapy process study. *Schizophr. Res.* **271**, 179–185 (2024). 10.1016/j.schres.2024.07.024 39032430

[8] Michalska Da Rocha, B., Rhodes, S., Vasilopoulou, E. & Hutton, P. Loneliness in Psychosis: A Meta-analytical Review. *Schizophr. Bull.* **44**, 114–125 (2018). 10.1093/schbul/sbx036 PMC5768045 28369646

[9] Catalano, L. T. & Green, M. F. Social Motivation in Schizophrenia: What’s Effort Got to Do With It? *Schizophr. Bull.* **49**, 1127–1137 (2023). 10.1093/schbul/sbad090 PMC10483329 37354079

[10] Engel, L. et al. The cost-effectiveness of a novel online social therapy to maintain treatment effects from first-episode psychosis services: results from the horyzons randomized controlled trial. *Schizophr. Bull.* **50**, 427–436 (2024). 10.1093/schbul/sbad071 PMC10919787 37261464

[11] Gafoor, R. et al. Effect of early intervention on 5-year outcome in non-affective psychosis. *Br. J. Psychiatry* **196**, 372–376 (2010). 10.1192/bjp.bp.109.066050 20435962

[12] Zhou, J., Lamichhane, B., Ben-Zeev, D., Campbell, A. & Sano, A. Predicting psychotic relapse in schizophrenia with mobile sensor data: routine cluster analysis. *JMIR Mhealth Uhealth* **10**, e31006 (2022). 10.2196/31006 PMC9039818 35404256

[13] Montaner-Ferrer, M. J., Gadea, M. & Sanjuán, J. Cognition and social functioning in first episode psychosis: A systematic review of longitudinal studies. *Front. Psychiatry* **14**, 1055012 (2023). 10.3389/fpsyt.2023.1055012 PMC10025326 36950257

[14] Barnett, J. H. et al. Visuospatial learning and executive function are independently impaired in first-episode psychosis. *Psychol. Med.* **35**, 1031–1041 (2005). 10.1017/s0033291704004301 16045069

[15] O’Connell, N. et al. Early intervention in psychosis services: a systematic review and narrative synthesis of the barriers and facilitators to implementation. *Eur. Psychiatr.* **65**, e2 (2022). 10.1192/j.eurpsy.2021.2260 PMC8792869 34913421

[16] Wenzel, J. et al. Ecological momentary assessment (EMA) combined with unsupervised machine learning shows sensitivity to identify individuals in potential need for psychiatric assessment. *Eur. Arch. Psychiatry Clin. Neurosci.* **274**, 1639–1649 (2024). 10.1007/s00406-023-01668-w PMC11422424 37715784

[17] Alvarez-Jimenez, M. et al. The Horyzons project: a randomized controlled trial of a novel online social therapy to maintain treatment effects from specialist first-episode psychosis services. *World Psychiatry* **20**, 233–243 (2021). 10.1002/wps.20858 PMC8129860 34002511

[18] Harvey, P. D. et al. Capturing clinical symptoms with ecological momentary assessment: convergence of momentary reports of psychotic and mood symptoms with diagnoses and standard clinical assessments. *Innov. Clin. Neurosci.* **18**, 24–30 (2021). PMC8195558 34150360

[19] Eisner, E., Berry, N. & Bucci, S. Digital tools to support mental health: a survey study in psychosis. *BMC Psychiatry* **23**, 726 (2023). 10.1186/s12888-023-05114-y PMC10559432 37803367

[20] Del Piccolo, M. C. et al. Smartphone-based self-monitoring in first episode psychosis: mixed-methods study of barriers and facilitators to engagement. *J. Med. Internet Res.* **27**, e71989–e71989 (2025). 10.2196/71989 PMC12380402 40857702

[21] Barnett, I. et al. Relapse prediction in schizophrenia through digital phenotyping: a pilot study. *Neuropsychopharmacology* **43**, 1660–1666 (2018). 10.1038/s41386-018-0030-z PMC6006347 29511333

[22] Wang, R. et al. On Predicting Relapse in Schizophrenia using Mobile Sensing in a Randomized Control Trial. in *2020 IEEE International Conference on Pervasive Computing and Communications (PerCom**)* 1–8 (IEEE, Austin, TX, USA, 2020). 10.1109/PerCom45495.2020.9127365.

[23] Bebbington, P. & Kuipers, L. The predictive utility of expressed emotion in schizophrenia: an aggregate analysis. *Psychol. Med.* **24**, 707–718 (1994). 10.1017/s0033291700027860 7991753

[24] Anderson, Z., Gupta, T., Revelle, W., Haase, C. M. & Mittal, V. A. Alterations in emotional diversity correspond with increased severity of attenuated positive and negative symptoms in the clinical high-risk syndrome. *Front. Psychiatry* **12**, 1–10 (2021). 10.3389/fpsyt.2021.755027 PMC8732994 35002795

[25] Kay, S. R., Fiszbein, A. & Opler, L. A. The positive and negative syndrome scale (PANSS) for schizophrenia. *Schizophr. Bull.* **13**, 261–276 (1987). 10.1093/schbul/13.2.261 3616518

[26] Hamilton, M. A rating scale for depression. *J. Neurol., Neurosurg., Psychiatry* **23**, 56–62 (1960). 10.1136/jnnp.23.1.56 PMC495331 14399272

[27] Pedersen, G. & Karterud, S. The symptom and function dimensions of the Global Assessment of Functioning (GAF) scale. *Compr. Psychiatry* **53**, 292–298 (2012). 10.1016/j.comppsych.2011.04.007 21632038

[28] Cornblatt, B. A. et al. Preliminary findings for two new measures of social and role functioning in the prodromal phase of schizophrenia. *Schizophr. Bull.* **33**, 688–702 (2007). 10.1093/schbul/sbm029 PMC2526147 17440198

[29] First, M. B., Williams, J. B. W., Karg, R. S. & Spitzer, R. L. Structured Clinical Interview For DSM-5® Disorders - Research Version (SCID-5 for DSM-5, Research Version; SCID-5-RV). (American Psychiatric Association, Arlington, VA, 2015).

[30] Chawla, N. V., Bowyer, K. W., Hall, L. O. & Kegelmeyer, W. P. SMOTE: synthetic minority over-sampling technique. *JAIR* **16**, 321–357 (2002).

