# Supplementary material for: Using smartphone-based assessment of social experience to predict relapse in first-episode psychosis
Source: Schizophrenia (Heidelb). 2026 Jul 17;12(1):75. doi: 10.1038/s41537-026-00786-3 (PMC13601519; doi:10.1038/s41537-026-00786-3)
Supplement: Supplementary file 1 [file 41537_2026_786_MOESM1_ESM.docx]

**Supplementary Note 1:**

Data from the 7-, 15-, and 30-day post-baseline assessment windows were split into training (70%) and testing (30%) sets. To ensure sufficient data for model training, participants were required to have completed a minimum number of assessments (at least one for 7 days; one or three for 15 days; and one, three, or five for 30 days). For each participant, average daily social contact and social experience scores, together with baseline sociodemographic variables, were used as model inputs.

Because relapse within 12 months was relatively infrequent (ratio of no relapse to relapse ≈ 4:1), the Synthetic Minority Oversampling Technique (SMOTE)^1^ was applied to the training set to address class imbalance. Models were developed using gradient-boosted decision trees (CatBoost), with hyperparameters optimized using the Optuna^2^ framework with 5-fold cross-validation on the training set to improve robustness.

Model performance was evaluated on the testing set using balanced accuracy, receiver operating characteristic (ROC) curves, and the area under the ROC curve (AUC). The optimal probability threshold was determined by maximizing the Youden index. Variability in AUC and balanced accuracy estimates was assessed using 1,000 bootstrap resamples to obtain 95% confidence intervals (CIs); the models were considered significantly better than chance if the 95% CI excluded 0.50. Comparisons of AUCs were performed using the DeLong test, and one-tailed binomial tests were used to compare balanced accuracy between models and against chance.

All preprocessing and modelling were conducted in Python using scikit-learn (v1.4.0), CatBoost (v1.2.8), and Optuna (v4.3.0). Statistical analyses were conducted in R (v4.4.3).

1.	Chawla, N. V., Bowyer, K. W., Hall, L. O. & Kegelmeyer, W. P. SMOTE: Synthetic Minority Over-sampling Technique. jair 16, 321–357 (2002).

2.	Akiba, T., Sano, S., Yanase, T., Ohta, T. & Koyama, M. Optuna: A Next-generation Hyperparameter Optimization Framework. in Proceedings of the 25th ACM SIGKDD International Conference on Knowledge Discovery & Data Mining 2623–2631 (ACM, Anchorage AK USA, 2019). doi:10.1145/3292500.3330701.

**Supplementary Table 1**. Univariate associations between being alone (vs. with others) and relapse within 4, 8, and 12 months, with sensitivity analyses using data from 7-, 15- and 30-day windows after baseline.

|  |  | Relapse | | |
| --- | --- | --- | --- | --- |
|  |  | Within 4 months | Within 8 months | Within 12 months |
|  |  | OR  95% CI | OR  95% CI | OR  95% CI |
| 7 days of smartphone data | Momentary social contact | 1.83*  (1.08, 3.11) | 1.57*  (1.00, 2.46) | 1.45*  (1.00, 2.11) |
|  | Daily social contact | 4.16***  (2.13, 8.12) | 3.14***  (1.65, 5.97) | 2.75**  (1.49, 5.06) |
| 15 days of smartphone data | Momentary social contact | 1.75*  (1.01, 3.05) | 1.60*  (1.01, 2.54) | 1.65*  (1.12, 2.43) |
|  | Daily social contact | 3.61***  (1.96, 6.65) | 2.59**  (1.40, 4.79) | 2.68**  (1.50, 4.78) |
| 30 days of smartphone data | Momentary social contact | 1.86  (1.00, 3.45) | 1.81*  (1.11, 2.94) | 1.90**  (1.26, 2.84) |
|  | Daily social contact | 3.55***  (1.81, 6.98) | 2.57**  (1.31, 5.04) | 2.80**  (1.48, 5.26) |

* p < 0.05
** p < 0.01
*** p < 0.001

**Supplementary Table 2**. Univariate associations between social experience and relapse within 4, 8, and 12 months, with sensitivity analyses using data from 7-, 15- and 30-day windows after baseline.

|  |  | Relapse | | |
| --- | --- | --- | --- | --- |
|  |  | Within 4 months | Within 8 months | Within 12 months |
|  |  | OR  95% CI | OR  95% CI | OR  95% CI |
| 7 days of smartphone data | Momentary social experience | 0.83***  (0.76, 0.90) | 0.88**  (0.81, 0.96) | 0.91*  (0.84, 0.98) |
|  | Daily social experience | 0.83***  (0.77, 0.90) | 0.89**  (0.82, 0.97) | 0.90**  (0.84, 0.97) |
| 15 days of smartphone data | Momentary social experience | 0.81***  (0.75, 0.88) | 0.87**  (0.80, 0.94) | 0.90**  (0.83, 0.97) |
|  | Daily social experience | 0.83***  (0.77, 0.89) | 0.89**  (0.82, 0.96) | 0.90**  (0.84, 0.97) |
| 30 days of smartphone data | Momentary social experience | 0.83***  (0.77, 0.90) | 0.88**  (0.82, 0.96) | 0.90**  (0.84, 0.96) |
|  | Daily social experience | 0.84***  (0.78, 0.91) | 0.90**  (0.83,0.97) | 0.90**  (0.84, 0.97) |

* p < 0.05
** p < 0.01
*** p < 0.001

**Supplementary Table 3**. Associations between social experience and relapse within 4, 8, and 12 months, with sensitivity analyses using data from 7-, 15- and 30-day windows after baseline. Analyses were adjusted for age, gender, ethnicity, employment status, education, and student status.

|  |  | Relapse | | |
| --- | --- | --- | --- | --- |
|  |  | Within 4 months | Within 8 months | Within 12 months |
|  |  | OR  95% CI | OR  95% CI | OR  95% CI |
| 7 days of smartphone data | Momentary social experience | 0.82***  (0.75, 0.89) | 0.88*  (0.80, 0.97) | 0.92*  (0.85, 0.99) |
|  | Daily social experience | 0.82***  (0.75, 0.90) | 0.89*  (0.81, 0.99) | 0.91*  (0.84, 0.98) |
| 15 days of smartphone data | Momentary social experience | 0.80***  (0.74, 0.86) | 0.87**  (0.80, 0.95) | 0.90*  (0.84, 0.98) |
|  | Daily social experience | 0.82***  (0.76, 0.89) | 0.90*  (0.82, 0.99) | 0.92*  (0.84, 0.99) |
| 30 days of smartphone data | Momentary social experience | 0.82***  (0.76, 0.89) | 0.89**  (0.82, 0.97) | 0.90**  (0.84, 0.97) |
|  | Daily social experience | 0.84***  (0.77, 0.91) | 0.91  (0.83, 1.00) | 0.91*  (0.84, 0.99) |

* p < 0.05
** p < 0.01
*** p < 0.001

**Supplementary Table 4**.

|  | Number of assessments | Sample size | Validation accuracy | Validation AUC | Training accuracy | Training AUC |
| --- | --- | --- | --- | --- | --- | --- |
| 7 days data collection | 1 | 219 | 0.715  [0.554, 0.855] | 0.707  [0.566, 0.845] | 0.699  [0.600, 0.793] | 0.747  [0.652, 0.837] |
| 15 days data collection | 1 | 221 | 0.715  [0.571, 0.845] | 0.768  [0.650, 0.870] | 0.910  [0.845, 0.967] | 0.959  [0.924, 0.989] |
|  | 3 | 193 | 0.788  [0.634, 0.829] | 0.772  [0.667, 0.871] | 0.792  [0.653, 0.915] | 0.816  [0.654, 0.955] |
| 30 days data collection | 1 | 225 | 0.722  [0.611, 0.824] | 0.754  [0.618, 0.871] | 0.717  [0.623, 0.808] | 0.808  [0.611, 0.824] |
|  | 3 | 209 | 0.745  [0.599, 0.892] | 0.807  [0.667, 0.927] | 0.785  [0.696, 0.870] | 0.890  [0.829, 0.944] |
|  | 5 | 189 | 0.790  [0.721, 0.860] | 0.829  [0.721, 0.929] | 0.727  [0.642, 0.800] | 0.838  [0.747, 0.921] |

**Supplementary Table 5.** Associations between sleep, social experience and social contact using data from 7-, 15-, and 30-day windows after baseline.

|  |  | Momentary social experience | Momentary social contact | Daily social experience | Daily social contact |
| --- | --- | --- | --- | --- | --- |
|  |  | Coef  95% CI | OR  95% CI | Coef  95% CI | OR  95% CI |
| 7 days of smartphone data | Did you have trouble falling asleep? | -0.47  (-0.98, 0.05) | 0.89  (0.59, 1.34) | -0.47  (-0.97, 0.02) | 1.00  (0.50, 1.99) |
|  | Do you feel you had enough sleep? | 0.71**  (0.22, 1.19) | 1.06  (0.73, 1.56) | 0.67**  (0.20, 1.13) | 0.68  (0.20, 1.13) |
|  | How many hours did you sleep? | 0.05  (-0.07, 0.18) | 1.08  (0.98, 1.19) | 0.16**  (0.04, 0.27) | 0.97  (0.83, 1.13) |
| 15 days of smartphone data | Did you have trouble falling asleep? | -0.58**  (-0.93, -0.22) | 0.97 (0.72, 1.32) | -0.35*  (-0.69, -0.00) | 0.98  (0.60, 1.61) |
|  | Do you feel you had enough sleep? | 0.54**  (-0.19, 0.89) | 0.87  (0.65, 1.15) | 0.65***  (0.31, 0.98) | 0.76  (0.47, 1.25) |
|  | How many hours did you sleep? | 0.02  (-0.07, 0.10) | 1.05  (0.98, 1.13) | 0.10*  (0.02, 0.18) | 0.95  (0.85, 1.07) |
| 30 days of smartphone data | Did you have trouble falling asleep? | -0.39**  (-0.67, -0.11) | 0.90  (0.71, 1.15) | -0.33*  (-0.60, -0.06) | 0.91  (0.62, 1.34) |
|  | Do you feel you had enough sleep? | 0.68***  (0.42, 0.95) | 0.99  (0.79, 1.23) | 0.40**  (0.14, 0.65) | 1.07  (0.73, 1.58) |
|  | How many hours did you sleep? | 0.00  (-0.06, 0.07) | 1.07*  (1.01, 1.13) | 0.03  (-0.03, 0.10) | 0.98  (0.90, 1.07) |

* p < 0.05
** p < 0.01
*** p < 0.001

**Supplementary Table 6**. Associations between social experience and relapse within 4, 8, and 12 months, with sensitivity analyses using data from 7-, 15- and 30-day windows after baseline. Analyses were adjusted for age, gender, ethnicity, employment status, education, student status, and sleep.

|  |  | Relapse | | |
| --- | --- | --- | --- | --- |
|  |  | Within 4 months | Within 8 months | Within 12 months |
|  |  | OR  95% CI | OR  95% CI | OR  95% CI |
| 7 days of smartphone data | Momentary social experience | 0.83***  (0.77, 0.90) | 0.89**  (0.82, 0.98) | 0.93  (0.86, 1.00) |
|  | Daily social experience | 0.85***  (0.78, 0.92) | 0.92  (0.84, 1.00) | 0.93  (0.86, 1.00) |
| 15 days of smartphone data | Momentary social experience | 0.88**  (0.81, 0.96) | 0.87**  (0.80, 0.95) | 0.91*  (0.85, 0.98) |
|  | Daily social experience | 0.92  (0.84, 1.00) | 0.90*  (0.82, 0.99) | 0.93  (0.86, 1.01) |
| 30 days of smartphone data | Momentary social experience | 0.83***  (0.77, 0.90) | 0.90**  (0.83, 0.97) | 0.91**  (0.85, 0.97) |
|  | Daily social experience | 0.85**  (0.78, 0.93) | 0.92  (0.83, 1.01) | 0.92*  (0.85, 0.99) |

* p < 0.05
** p < 0.01
*** p < 0.001

**Supplementary Table 7**. Associations between being alone (vs. with others) and relapse within 4, 8, and 12 months, with sensitivity analyses using data from 7-, 15- and 30-day windows after baseline. Analyses were adjusted for age, gender, ethnicity, education level, employment, student status and sleep.

|  | | Relapse | | |
| --- | --- | --- | --- | --- |
|  |  | Within 4 months | Within 8 months | Within 12 months |
|  |  | OR  95% CI | OR  95% CI | OR  95% CI |
| 7 days of smartphone data | Daily social contact | 3.21**  (1.45, 7.10) | 2.65**  (1.28, 5.48) | 2.17*  (1.03, 4.55) |
|  | Momentary social contact | 2.23  (0.95, 5.22) | 1.57  (0.84, 2.95) | 1.41  (0.88, 2.27) |
| 15 days of smartphone data | Daily social contact | 2.37*  (1.18, 4.76) | 1.84  (0.93, 3.65) | 1.83  (0.90, 3.72) |
|  | Momentary social contact | 1.83  (0.83, 4.01) | 1.47  (0.82, 2.63) | 1.59*  (1.01, 2.52) |
| 30 days of smartphone data | Daily social contact | 2.14  (0.94, 4.88) | 1.90  (0.90, 4.01) | 1.82  (0.85, 3.91) |
|  | Momentary social contact | 1.81  (0.85, 3.87) | 1.57  (0.93, 2.64) | 1.79*  (1.14, 2.81) |

*Key: * p < 0.05	** p < 0.01	*** p < 0.001*

**Supplementary Table 8.** Predictors included in the regression and machine learning models.

| **Variable name** | **Construct** | **Source** | **Question** | **Coding or scoring** | **Included in regression models** | **Included in machine learning models** |
| --- | --- | --- | --- | --- | --- | --- |
| *age* | Age | Baseline sociodemographic assessment | How old are you? | Continuous, years | Yes, covariate | Yes |
| *gender* | Gender | Baseline sociodemographic assessment | What is your gender? | Binary  [Male, Female] | Yes, covariate | Yes |
| *ethnicity* | Ethnicity | Baseline sociodemographic assessment | What is your ethnicity? | Categorical  [White, Black, Asian, Other] | Yes, covariate | Yes |
| *employment* | Employment status | Baseline sociodemographic assessment | Are you currently working? | Binary  [Not working, Working] | Yes, covariate | Yes |
| *education* | Student status | Baseline sociodemographic assessment | Are you currently in education? | Binary  [Not in education, In education] | Yes, covariate | Yes |
| *education_level* | Highest education level | Baseline smartphone assessment | What is your highest level of education? | Categorical  [Less than high school, Professional training, High school, University, Post-graduate] | Yes, covariate | Yes |
| *withyounow_alone* | Momentary social contact | Smartphone-based assessment | Who are you with right now? | Binary  [With others, Nobody/Alone] | Yes | Yes |
| *hours24_alone* | Daily social contact | Smartphone-based assessment | Who have you been with over the last 24 hours? | Binary  [With others, Nobody/Alone] | Yes | Yes |
| *now_social* | Momentary social experience | Smartphone-based assessment | Composite score based on ratings of the current social context: relaxing, enjoyable, stressful, upsetting, and preference not to be in that context | Two positive items were retained as scored; three negative items were reverse scored. All five items were summed; higher scores indicate more positive momentary social experience. | Yes | Yes |
| *hours24_social* | Daily social experience | Smartphone-based assessment | Composite score based on ratings of social context over the previous 24 hours: relaxing, enjoyable, stressful, upsetting, and preference not to be in that context | Two positive items were retained as scored; three negative items were reverse scored. All five items were summed; higher scores indicate more daily momentary social experience. | Yes | Yes |
